# Fever-Range Hyperthermia vs. Hypothermia Effect on Cancer Cell Viability, Proliferation and HSP90 Expression

**DOI:** 10.1371/journal.pone.0116021

**Published:** 2015-01-30

**Authors:** Dimitra Kalamida, Ilias V. Karagounis, Achilleas Mitrakas, Sofia Kalamida, Alexandra Giatromanolaki, Michael I. Koukourakis

**Affiliations:** 1 Department of Radiotherapy/Oncology, Democritus University of Thrace, Alexandroupolis, 68100, Greece; 2 Department of Pathology, Democritus University of Thrace, Alexandroupolis, 68100, Greece; Ludwig-Maximilians University, GERMANY

## Abstract

**Purpose:**

The current study examines the effect of fever-range hyperthermia and mild hypothermia on human cancer cells focusing on cell viability, proliferation and HSP90 expression.

**Materials and Methods:**

A549 and H1299 lung carcinoma, MCF7 breast adenocarcinoma, U87MG and T98G glioblastoma, DU145 and PC3 prostate carcinoma and MRC5 normal fetal lung fibroblasts cell lines were studied. After 3-day exposure to 34°C, 37°C and 40°C, cell viability was determined. Cell proliferation (ki67 index), apoptosis (Caspase 9) and HSP90 expression was studied by confocal microscopy.

**Results:**

Viability/proliferation experiments demonstrated that MRC5 fibroblasts were extremely sensitive to hyperthermia, while they were the most resistant to hypothermia. T98G and A549 were thermo-tolerant, the remaining being thermo-sensitive to a varying degree. Nonetheless, as a universal effect, hypothermia reduced viability/proliferation in all cell lines. Hyperthermia sharply induced Caspase 9 in the U87MG most thermo-sensitive cell line. In T98G and A549 thermo-tolerant cell lines, the levels of Caspase 9 declined. Moreover, hyperthermia strongly induced the HSP90 levels in T98G, whilst a sharp decrease was recorded in the thermo-sensitive PC3 and U87MG cell lines. Hyperthermia sensitized thermo-sensitive cancer cell lines to cisplatin and temozolomide, whilst its sensitizing effect was diminished in thermo-tolerant cell lines.

**Conclusions:**

The existence of thermo-tolerant and thermo-sensitive cancer cell lines was confirmed, which further encourages research to classify human tumor thermic predilection for patient stratification in clinical trials. Of interest, mild hypothermia had a universal suppressing effect on cancer cell proliferation, further supporting the radio-sensitization hypothesis through reduction of oxygen and metabolic demands.

## Introduction

Cancer cells, similarly to any other cell and living system, respond to mild changes of external temperature by activating homeostatic biological mechanisms, in an attempt to sustain a tolerant intracellular environment and prevent death. Temperatures above 41°C are toxic both to the tumor vasculature and to the cancer cells themselves, and are used to heat tumors (Oncothermic Hyperthermia) aiming to suppress their growth, achieve regression or to sensitize them to radiotherapy and chemotherapy [1]. Randomized trials have demonstrated significant improvement of local control rates in sarcomas applying regional hyperthermia combined with chemotherapy [2].

Nevertheless, temperatures below 41°C, at the so called fever-range hyperthermia, have also a direct effect on cancer cell and tissue biology, sensitizing tumors to radiotherapy and chemotherapy. Whole body hyperthermia between 38–40°C has been used in the treatment of wide-spread metastatic tumors in combination with chemotherapy [3, 4]. Increased blood flow that allows increased tumor oxygenation and chemotherapy availability is probably one of the main mechanisms of synergism [5, 6]. Protein damage is also a critical effect of temperatures above 39°C, but the exact pathways of cell killing remain elusive [7]. Inhibition of homologous recombination has been also proposed as a mechanism of tumor chemosensitization [8].

In addition, mild hyperthermia as a complementary therapy to cancer immune-therapy has been presented by several preclinical and clinical studies, by improving antitumor immune responses. The hyperthermia- induced improved immune response includes HSPs generation, antigen presenting cells activation and lymphocytes trafficking changes [9]. Furthermore, additional evidence indicates that physiological responses to induced hyperthermia affects the microenvironment of the tumor most likely by a mechanism involving temperature-sensitive check-points regulating tumor vascular perfusion, lymphocyte trafficking, inflammatory cytokine expression, tumor metabolism, and last but not least, both adaptive and innate immune action [10]. Increased activity of natural killer cells against colon tumor cells has been verified in mice when temperature was increased at 39.5°C [11].

The Heat-Shock Proteins (HSPs) are sharply up-regulated under hyperthermic conditions, as their higher affinity to accumulating unfolded proteins releases the HSF-1 transcription factor bound to HSPs which enters the nuclei to initiate HSP gene transcription [11]. HSPs protect cells against heat-induced protein damage by their chaperon activity. However, prolonged heating may result in HSP levels regression to normal levels. On the contrary, high temperatures above a threshold may inhibit HSP synthesis, which favors cell death [12]. In more details, the Hsp90/Hsp70-based chaperone machinery has been demonstrated to control the signaling of protein function, trafficking and turnover. It has been also recently suggested, that Hsp90 and Hsp70 regulate the processing of damaged and abnormal proteins for degradation via the ubiquitin-proteasome pathway. The study of HSP90 in particular, is highly important, given that, thus far scientific interest was focused on the regulation of its’ typical client proteins, but recent data suggest that HSP90 interacts dynamically with various proteins, which are not classic HSP90 clients. Therefore, HSP90’s role in signaling and regulating the quality control and the fate of damaged proteins is extremely important. [13]

In any case, cancer cell response to hyperthermia may depend on both temperature levels and exposure times, additional ambient conditions and, certainly, on the cell type under investigation. In the current study, we examined the effect of fever-range hyperthermia on a wide range of cancer cells, providing evidence that its effect on viability and proliferation is cell-dependent. Hypothermia, a far less studied condition in cancer cell systems, was also investigated. The effect of hyper- and hypothermia on HSP90 expression was further examined. Finally, hyperthermic chemosensitization was examined in two glioblastoma cell lines (T98G and U87MG) and two lung cancer cell lines (A549 and H1299). T98G and A549, the only thermo-tolerant cell lines were examined in comparison with two representative, thermo-sensitive cell lines of the same tumor type, U87MG and H1299, respectively, with the appropriate chemotherapy drugs for each type of cancer, together with mild hyperthermia, in order to examine a possible sensitisation or resistance.

## Materials and Methods

### Cell cultures

A549 (human lung adenocarcinoma, CLS GmbH, Germany), H1299 (human non-small cell lung carcinoma, ATCC), MCF7 (human breast adenocarcinoma, CLS GmbH, Germany), U87MG (human glioblastoma-astrocytoma, CLS GmbH, Germany), DU145 (human prostate carcinoma, CLS GmbH, Germany), PC3 (human prostate adenocarcinoma, CLS GmbH, Germany) and MRC5 (human fetal lung fibroblasts, CLS GmbH, Germany) cell lines were cultured using DMEM basal medium (31885-023, Gibco) and T98G (human glioblastoma multiforme, ATCC) cell line was grown in MEM basal medium (10370-047, Gibco). Both basal culture mediums were supplemented with 10% FBS (FB-1000/500, Biosera), 100 units/ml Penicillin and 100μg/ml Streptomycin (15140-122, Gibco) and 2mM L-Glutamine (25030, Gibco). Cells were maintained at standard conditions, 37°C, 5% CO2 in humidified atmosphere and were used upon reaching 70–90% confluency.

### Proliferation Assay

The ability of cell proliferation under different incubation temperatures was tested by a proliferation assay. Cells were seeded in 96-well plates at a density of 1000 cells/well, the plates were incubated at 37°C for 3h to facilitate adherence and normal growth and then were placed at 34°C, 37°C and 40°C, respectively, for three consecutive days. Proliferations assays for all the examined cell lines and growth temperatures were performed simultaneously. Cell proliferation measurements were performed in a 24h interval using the AlamarBlue Cell Viability Reagent (DAL1100, Invitrogen) measured at 540nm excitation and 590nm emission wavelengths, in a FLUOstar Omega microplate reader (BMG LABTECH). The AlamarBlue assay (DAL1100, Invitrogen) is a reliable method for cell viability [14]. This assay, by using the metabolic activity of cells to reduce resazurin (oxidized form; 7-hydroxy-3H-phenoxazin-3-1-10-oxide) to resorufin, quantifies the number of cells with active mitochondria, since resazurin reduction is performed by mitochondrial enzymes [15]. The experiments were performed three times in order to confirm the significance of the results.

### Western Blot analysis

Glioblastoma cell lines (U87MG and T98G) were cultured under standard conditions, as previously mentioned. These cell lines were similarly incubated at 34°C and 40°C for 72h. Cells were lysed using a sucrose lysis buffer supplemented with a protease and phosphatase inhibitor cocktail (Cell Signaling). Protein concentrations were measured based at the BCA protein assay kit (Thermo Scientific Pierce, USA). Specific rabbit polyclonal primary antibodies were used for western blot analysis, anti-HSP90 (1:1000; ab13495, Abcam) and anti-Caspase9 (1:1000; ab47537, Abcam).

Whole fraction samples were separated on discontinuous SDS gels using 10% separating and 5% stacking gels. Forty micrograms of the cell extracts were loaded on the gel. Immunoblotting was performed utilizing PVDF-PSQ membranes (Millipore Corp.). Following a blocking step with 5% non-fat dry milk in 150 mM NaCl, 10 mM Tris, pH 7.5 containing 0.1% (v/v) Tween 20 (TBS-T) at room temperature (RT) for 2h, the membranes were hybridized overnight at 4°C with the primary antibodies. The membranes were then hybridized for 2h at 37°C with the secondary antibody, goat polyclonal to rabbit IgG (H+L)-HRP (1:3.000, Biorad, 1706515, USA) and finally developed in Amersham ECL Western blotting detection reagents and analysis system (RPN2209, GE Healthcare) utilizing Chemidoc MP Imaging System (Biorad, USA).

### Hyperthermic chemosensitization experiments

In chemosensitization experiments, 1000 cells/well were plated in a 96-well plate in the appropriate culture medium. Cells were incubated with clinically established drugs; lung cancer cell lines: A549 and H1299 with 100 μΜ of Cisplatin and glioblastoma cell lines: T98G and U87MG with 500 μΜ of temozolomide. The cells, simultaneously with the drug treatment, were incubated for 24h at 37ºC and 40ºC, respectively, while cells’ viability was assessed after 24h incubation period by AlamarBlue assay and the cells survival percentages (%) were calculated. Statistical analysis by the 2way ANOVA test (n≥ 10 measurements for each group, ** p = 0.0037 for 5a p = 0.0097 for 5b, which is statistically significant in both cases) and graph presentation has been performed using the GraphPad Prism Version 5.01 statistical package (GraphPad Software Inc., USA). The experiments were performed three times in order to confirm the significance of the results.

### Confocal immunofluorescence and Image analysis

For immunofluorescence staining, cells were grown on No. 1.5 glass coverslips, fixed in 3.7% paraformaldehyde/PBS pH 7.4 for 20 min at 37°C and then permeabilized in PBS/0.1% v/v Triton X-100 pH 7.4 for 5 min at room temperature. In addition, cells were blocked in PBS/5% w/v BSA pH 7.4 for 20 min and stained with various primary antibodies: anti-ki67 mouse monoclonal (1:150; DAKO) anti-Caspase9 rabbit polyclonal (1:100; Abcam), anti-HSP90 rabbit polyclonal (1:100; Abcam), for 1 h at RT. Cells were washed in PBS pH 7.4, incubated with appropriate CF 488 and 564 secondary antibodies at RT and DNA was counterstained with Hoechst 33342 (1 µg/ml; Sigma-Aldrich). After final washes coverslips were mounted in homemade Mowiol mounting medium. Imaging was performed on a customized Andor Revolution Spinning Disk Confocal System built around a stand (IX81; Olympus) with a 60x lens and a digital camera (Andor Ixon+885) (CIBIT Facility, MBG-DUTH). Image acquisition was performed in Andor IQ 2 software. Optical sections were recorded every 0.3 µm. All confocal microscopy images presented in this work are 2D maximum intensity projections of z-stack images (ImageJ 1.47v National Institute of Health, USA).

Image intensity analysis for the obtained data sets has been performed using ImageJ 1.47v (National Institute of Health, USA) software. Image processing macros have been custom developed in order to quantify the levels of the examined proteins (% Fluorescence Intensity) for Caspase 9 and HSP90 in the area of interest.

Image analysis and quantification of ki67 expression in the nucleus have been performed using custom developed macros in ImageJ software 1.47v (National Institute of Health, USA). A population of n cells (n≥ 20) has been analyzed, for ki67 quantification. The cells were categorized in three classes, according to their pixel units, representing ki67 expression levels. Class I was the lower class including cells with 1000–5000 pixels in the green channel (ki67 imaging) and Class III was the higher class including cells with more than 7501 pixels, while Class II included 5001–7500 values, respectively.

The two-dimensional (2D) average projection of z-stack images were quantified using a standard size square area where integrated intensity values have been measured. Statistical analysis by the 2way ANOVA test (n≥ 20 cells for each group, * p < 0.0001) and graph presentation has been performed using the GraphPad Prism Version 5.01a statistical package (GraphPad Software Inc., USA).

## Results

### Effect of hyper- and hypothermia on cell growth

**Figure 1:**
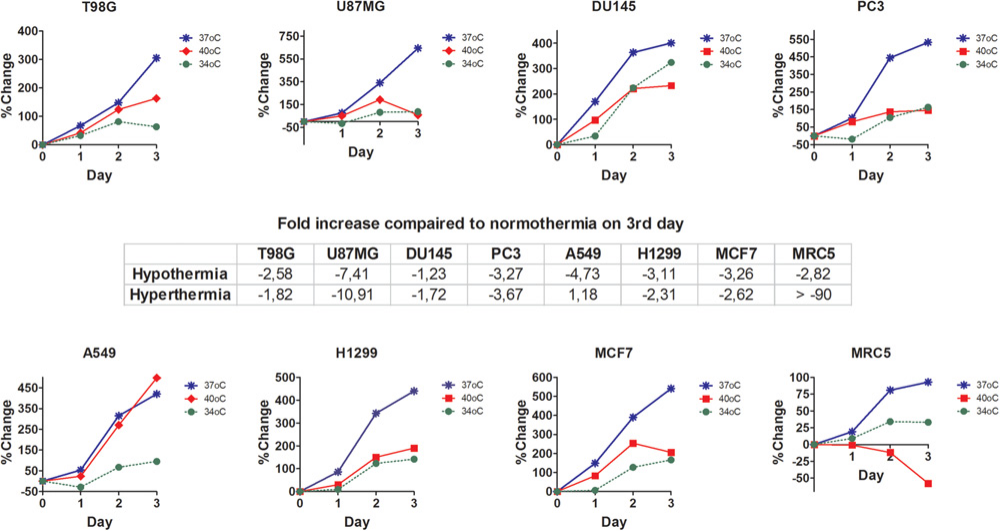
Cell proliferation studies with AlamrBlue after 3days in various incubation temperatures. % change of relative fluorescent units (RFUs) as recorded with the AlamarBlue assay, after 3 days of exposure of cells to hypothermia (34°C) or hyperthermia (40°C) compared to normothermia (37°C).

Following a 3-day incubation period under fever range hyperthermia, changes of cell proliferation/viability were strongly dependent upon each cell line (Fig. 1). The normal fibroblast cell line, MRC5, was extremely sensitive to hyperthermia, showing a prevalent cell death effect. U87MG glioblastoma cell line was also very sensitive to hyperthermia, which induced a 10-fold reduction in cell growth. The prostate DU147 and PC3, as well as the lung H1299 and breast MCF7 cancer cell lines were also thermo-sensitive. On the contrary, T98G glioblastoma and A549 lung cancer cell lines were thermo-tolerant, demonstrating a growth rate increase by 1.87 and 1.18 fold, respectively.

Hypothermia at 34°C, on the other hand, had a rather homogeneous effect, resulting in reduction of both the normal MRC5 fibroblasts and of all cancer cell lines viability (Fig. 1). This reduction ranged between 1.23 and 7.40 fold, in DU145 and U87MG cell lines, respectively, compared to control (37°C) cells, as calculated on the third day of incubation.

### Effect of hyper- and hypothermia on Ki67 proliferation index

**Figure 2:**
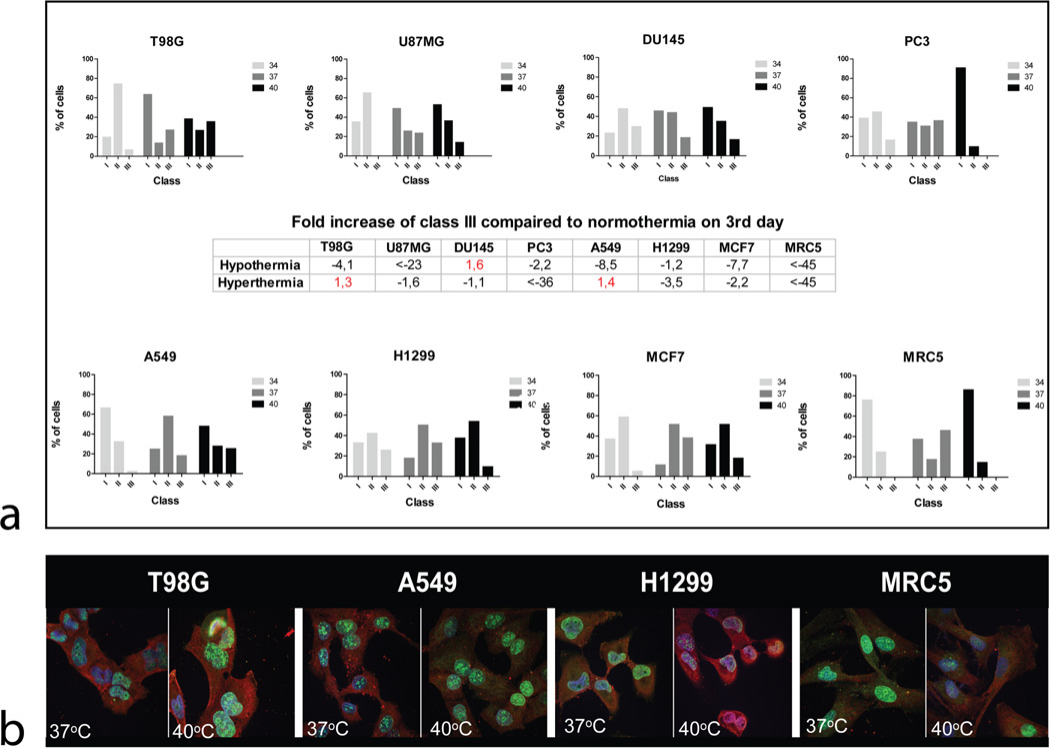
Confocal immunofluorescent microscopy images and automated quantification of ki67 proliferation marker in various cell lines. 2a: changes of Ki67 proliferation index class after 3-day exposure of cells to hypothermia (34°C) or hyperthermia (40°C) compared to normothermia (37°C). 2b: Representative confocal microcopy images showing nuclear Ki67 immunostaining changing intensity after exposure to hyperthermia (40°C).

Given that the cell proliferation/viability assessed by the AlamarBlue assay is a result of combined proliferation and death events, we further examined the cell proliferation using the ki67 proliferation index. After 3 days of cell incubation, hyperthermia resulted in an increased fraction of cells in class III, inT98G and A549 (1.3 and 1.4 fold respectively) cell lines compared to normothermia. This dropped by 1.1 to more than 45 fold in the rest of cell lines (Fig. 2a). Characteristic and representative confocal images of Ki67 nuclear staining and changes after exposure to hyperthermia are shown in Fig. 2b.

Hypothermia resulted in decrease of cell accumulation in the class III group by 1.2 up to more than 45 fold in all cell lines with the exception of DU145 prostate cancer cell line, where this was increased by 1.6 fold.

### Effect of hyper- and hypothermia on Caspase 9 levels

**Figure 3:**
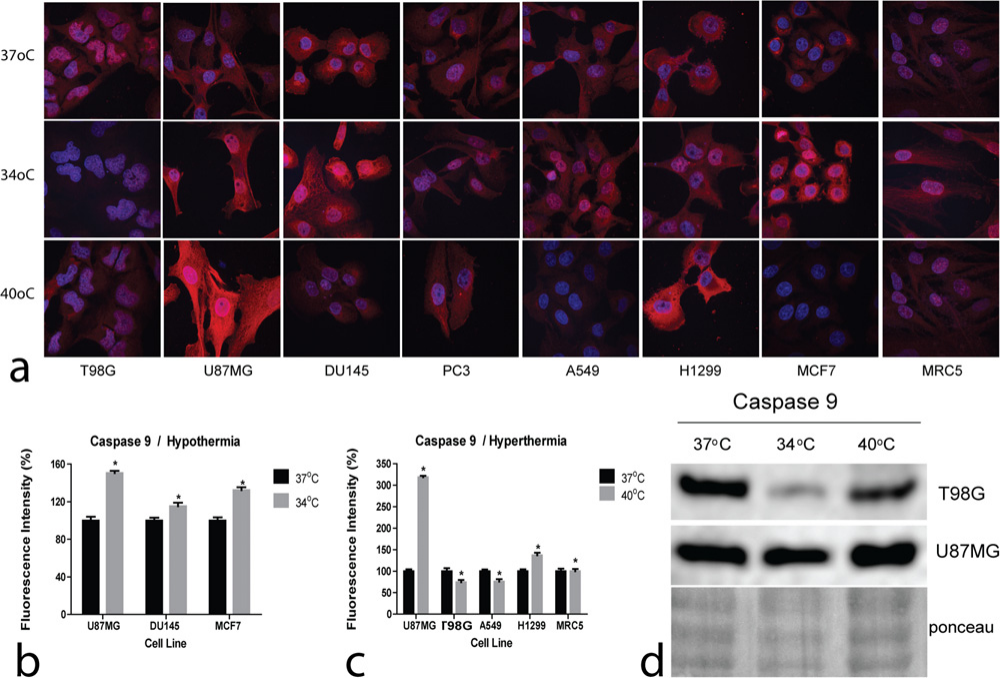
Confocal immunofluorescent microscopy and western blot images of Caspase9. 3a: Representative confocal microcopy images showing cytoplasmic Caspase 9 expression changing intensity after exposure to hypothermia (34°C) or hyperthermia (40°C) compared to normothermia (37°C) (magnification x60). 3b,c: Densitometry performed on confocal microcopy images of Caspase 9 immunostaining after exposure to hypothermia (34°C) or hyperthermia (40°C) compared to normothermia (37°C).

Confocal immunofluorescence images of Caspase 9 expression in cell lines following exposure to hyperthermia and hypothermia are presented in Fig. 3a. Plots of the fluorescence intensity changes following exposure to hyperthermia and hypothermia are presented in Fig. 3b and 3c, respectively.

Hyperthermia sharply induced Caspase 9 in the U87MG thermo-sensitive cell line, which also exhibited the most profound reduction of cell viability in Alamarblue experiments. Of interest, both T98G and A549 thermo-tolerant cell lines, Caspase 9 levels were reduced by hyperthermia, suggesting an apoptosis suppressing effect of hyperthermia in these cell lines. The most thermo-sensitive of all, MRC5 cell line, however, did not show increased Caspase 9 levels suggesting death by independent of Caspase 9 pathways.

Hypothermia induced Caspase 9 in U87MG, DU145 and MCF7 cells, in agreement with the reduced viability, demonstrated in Alamarblue experiments. In the rest of cell lines, Caspase 9 expression remained stable or it was reduced in the case of T98G cells, suggesting that if apoptosis pathways are activated by hypothermia these are Caspase 9 independent.

Western blot analysis performed in the thermo-tolerant T98G and the thermo-sensitive U87MG glioblastoma cell lines are presented in Fig. 3d, supporting the previously presented results. Hyperthermia induced Caspase 9 in the thermo-sensitive U87MG cell line, while this was reduced in the thermo-tolerant one, T98G. Hypothermia reduced Caspase 9 levels in the T98G cell line, while no evident change was noted in the U87MG one.

### Effect of hyper- and hypothermia HSP90 levels

**Figure 4:**
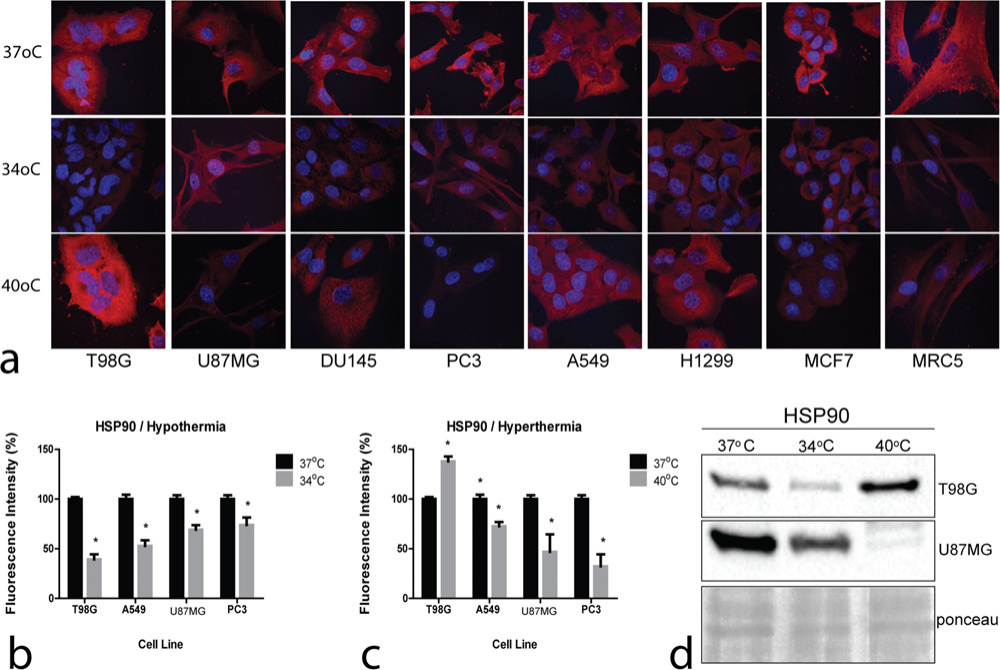
Confocal immunofluorescent microscopy and western blot images of HSP90. 4a: Representative confocal microcopy images showing cytoplasmic HSP90 expression changing intensity after exposure to hypothermia (34°C) or hyperthermia (40°C) compared to normothermia (37°C) (magnification x60). 4b,c: Densitometry performed on confocal microcopy images of HSP90 immunostaining. 4d: Western blot images of HSP90 expression in the thermo-tolerant T98G and the thermo-sensitive U87MG cell lines.

Representative confocal microscopy and immunofluorescence images of the effect of hyperthermia and of hypothermia on HSP90 on cell lines are shown in Fig. 4a. Plots of fluorescence intensity changes are presented in Fig. 4b and c.

Hyperthermia strongly increased the HSP90 levels in the T98G thermo-tolerant cell line, whilst a sharp drop was recorded in the thermo-sensitive PC3 and U87MG cell lines. On the other hand, the HSP90 levels were clearly reduced in all cell lines examined under hypothermia. Western blot analysis performed in the thermo-tolerant T98G and the thermo-sensitive U87MG glioblastoma cell lines are shown in Fig. 4d, confirming the results of confocal microscopy.

### Hyperthermic chemosensitization

**Figure 5:**
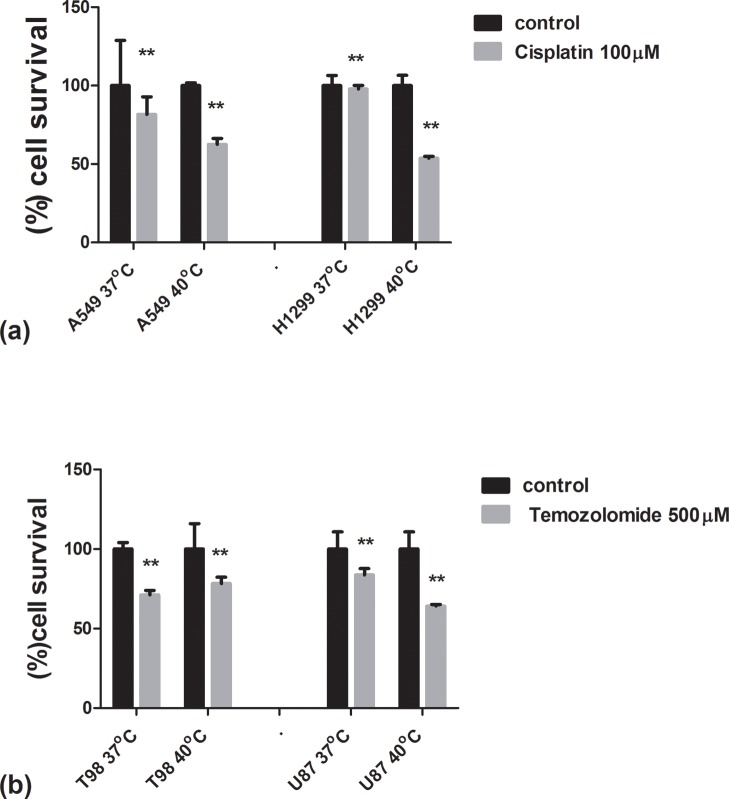
Hyperthermic chemosensitization experiments with Cisplatin and Temozolomide using the AlamarBlue assay. 5a: Viability of lung cancer cell lines A549 and H1299 after a 24h exposure to cisplatin under normothermic and fever range hyperthermic conditions. 5b: Viability of glioblastoma cell lines T98G and U87MG after a 24h exposure to temozolomide under normothermic and fever range hyperthermic conditions.

Exposure of the thermo-tolerant A549 and the thermo-sensitive H1299 lung cancer cell lines to cisplatin, the key drug used in the clinical practice for the treatment of lung cancer, showed that fever range hyperthermia strongly sensitized H1299 to the drug but its effect on the A549 cell line was minimal (Fig. 5a).

Exposure of the thermo-tolerant T98G and the U87MG thermo-sensitive glioblastoma cell lines to temozolomide, the only approved drug for the treatment of human glioblastoma, showed that hyperthermia at 40 ºC strongly sensitized the U87MG cell line to the drug, whilst no sensitizing effect was noted for the T98G one (Fig. 5b).

## Discussion

The effect of fever-range hyperthermia on normal and cancer cell biology and its eventual role and influence in cell sensitivity to chemotherapy and radiotherapy remains poorly understood, requiring further investigation. Mild hyperthermia has been reported to have an inhibitory effect on cell proliferation. In a previous study, however, Morrisey *et al* also reported a stimulatory effect of mild hyperthermia at 38°C on U87MG cell line that was sharply reversed at 40°C [16]. The conclusion made by these researchers regarding the differential response among cell lines to small temperature elevations is certainly important. The current study has lead as to the identification of two cell lines (T98G and A549) which seem to be resistant to the effect of hyperthermia at 40°C. The human A549 cell line has been previously reported to be resistant to thermal killing at 43–45°C compared to the U87MG cell line [17], but a differential response ranging from proliferation to cell killing at the well tolerated by the human body 40°C is new. Combinations of fever-range hyperthermia may, therefore, delay progression of metastatic disease in thermo-sensitive tumors with U87MG-like behaviour, while G2-M phase targeting drugs may prove critical to treat thermo-tolerant T98G-like tumors in combination with total body fever induction or local non-toxic heating.

On the other hand, the therapeutic role of hypothermia should not be underestimated and should be thoroughly examined in animal models, as about half of the examined cell lines demonstrated a 3–7 fold reduction of viability at 34°C. The current knowledge on its effect on cancer cell is limited. Hypothermia at 28°C seems to protect preferentially normal fibroblasts compared to cancer cells against 5-fluorouracil [18]. In our study, at 34°C, normal human fibroblasts suffered a reduced proliferation of an extent, however, quite limited compared to the majority of cancer cell lines. The reduced metabolism and oxygen consumption of tumors exposed to hypothermia may also be important in tumor radiosensitization [19], a hypothesis that has been also tested in the clinical practice [20]. The role of hypothermia in inhibiting cancer cell adhesion to endothelial cells and thus migration as shown by Zhang *et al* [21], provides an additional basis for further studies on the usage of hypothermia as a cancer therapy option.

We further examined whether the death effect induced by mild temperature changes in several cell lines is Caspase-9-mediated. The aspartic acid specific protease Caspase-9 is involved in the mitochondrial death pathway. Release of cytochrome c from mitochondria activates apaf-1 (apoptosome), which in its turn cleaves the pro-enzyme of Caspase-9 into its most active form. Nevertheless, cleavage is not essential for Caspase-9 apoptotic activity [22]. In our study, hypothermia and hyperthermia induced Caspase-9 in several sensitive cell lines like U87MG, DU145 and MCF7, in accordance with the reduced viability noted. In the rest of the cases, Caspase-9 expression remained stable, suggesting that if apoptotic pathways are activated by hypothermia these are Caspase 9 independent. For instance, AIF (apoptosis inducing factor) or Caspase-8 and 12 are alternative apoptotic Caspase-9 independent apoptotic pathways [23 ]. Of interest, in the T98G and A549 thermo-tolerant cell lines, Caspase-9 levels were reduced under fever-ranged hyperthermia, providing evidence for a pathway exploited by some cell lines to escape mitochondrial related apoptosis.

HSP90, on the other hand, is a heavy member of the HSP family, with an important role in tumor growth, being also linked with poor prognosis in breast cancer and other malignancies [24]. The HSPs are up-regulated under hyperthermic conditions [11], to protect cells against heat-induced protein damage by their chaperon activity. Several HSP90 inhibitors have been developed and have been demonstrated in *vivo* to be tumoristatic and to have a synergistic effect with chemotherapy and other target therapies [25]. The finding that hypothermia reduced the expression of HSP90 in all cell lines examined is interesting. Acting as a physical agent inhibitor of HSP90, hypothermia could have synergistic effect with chemotherapy, similarly to the chemical inhibitors. Of interest, hyperthermia enhanced HSP90 expression in the thermo-tolerant T98G cell line, which suggests a protective role of HSP90 in such cell lines flourishing under warm conditions.

Hyperthermia has been also studied in combination with chemotherapy drugs. It has been previously reported that simultaneous hyperthermia and cisplatin induced cell death in T leukemic cells by different molecular mechanisms, in that way the enhanced cisplatin-induced cytotoxicity by hyperthermia can be explained [26]. Similar data have been reported in a Phase I–II study aiming to evaluate the feasibility and severe toxicity of a combination of cisplatin, irradiation and hyperthermia, in the treatment of superficial cervical nodal metastases from head and neck cancer, where the feasibility of the combination of cisplatin, irradiation and local external microwave hyperthermia with an acceptable toxicity profile has been confirmed. Therefore, it was further suggested that the trimodal therapy deserves further assessment as a way to enhance the efficacy of irradiation in cases of nodal metastases from head and neck tumors [27]. In our study, experiments of simultaneous exposure of lung cancer and glioblastoma cell lines to fever range hyperthermia and clinically established chemotherapeutic drugs showed that sensitization conferred by hyperthermia mainly concerned the thermo-sensitive cell lines, whilst its sensitizing effect was drastically inferior in thermo-tolerant cell lines. This finding further supports the necessity to develop clinical methods able to identify thermo-sensitive tumors that would benefit the most if treated with combined hyperthermia and chemotherapy protocols. Whether thermo tolerant tumors might become sensitive to hyperthermic chemotherapy by blocking the HSP90 or relevant biological pathways remains an hypothesis for further experimentation.

It is concluded that cancer cells respond differentially to mild temperature changes, whether these are towards mild hypothermia or fever-range hyperthermia. Thermo-tolerant and thermo-sensitive cells lines have been identified to fever range hyperthermia, which encourages research to identify suitable methods for clinical grouping of human tumors according to their thermal predilection. Such characterization would allow clinical trials with non-toxic localized or total body hyperthermia in patients predicted to be sensitive to such therapies. Of interest, mild hypothermia had a suppressing effect on cell proliferation in all cells examined, suggesting that hypothermia would suppress tumor replication and metabolism in most human tumors, further supporting the radio-sensitization hypothesis through increased oxygenation by reducing oxygen and metabolic demands.
